# The Medical Services of the Forces during the War

**Published:** 1918-07-20

**Authors:** 


### The Hospital

The Workers' Newspaper of Administrative Medicine and Institutional Life, Administration, National Insurance and Health.

No. 1676, Vol. LXIV. SATURDAY, JULY 20, 1918. PRICE TWOPENCE

### THE MEDICAL SERVICES OF THE FORCES DURING THE WAR.

Owing to the war the hospitals of New York City are experiencing grave and constantly growing difficulty in maintaining working forces adequate to their needs. We give on another page some information concerning the position of affairs there and its difficulties, and hope the particulars of a system for the maintenance of the Medical Services of the Forces which has been evolved in England only during the war may prove helpful to our American Allies. By the experience of mistakes, many of them due to ignorance, the lesson has been learnt, at least in part. Efficiency lies in the use of medical students in a medical capacity. With conscription in force, combatant officers can be selected from suitable men already trained, and, it may be, already tested in the firing line; such a man can be made an officer by a stroke of the official pen. Such is not the case with the medical officer; a long period, five or six years, is necessary for his training.

At the beginning of the war the War Office encouraged the enlistment of medical students as combatants; the medical schools gave divided counsels, and many of the medical students, both junior and senior, joined as combatants. The example they set in fighting spirit, leadership, and disregard of danger has been a credit to the medical profession, but has not outweighed the disorganisation of the entry of medical students, and some reflection is even now in less well informed quarters thought to attach to those students who, under advice, persisted in their medical studies, joined the Forces as medical officers, and as such displayed courage equal to that of any combatants.

The wastage of a long war, and the needs of the civil population, at last impressed the authorities. In December, 1916, the Army Council issued instructions that fourth and fifth year medical students were not to be "called-up" if they produced certificates from the Dean of their medical school, and were enrolled in an Officers' Training Corps; medical students in any year, and not passed fit for general service, had their "calling-up" suspended; and fourth and fifth year students serving with the Colours were relegated to the Reserve in order to resume their medical studies. In less than a year it was necessary to extend these instructions, and third-year medical students were transferred to the Reserve. Within three months it was recognised that the supply for the Medical Services was still inadequate, and junior medical students who had passed the professional examination in chemistry, physics, and biology at the time of enlistment were transferred to the Reserve, and the authorities of the medical schools were urged by the Ministry of National Service to communicate with their students who were serving with the Colours, and impress upon them that they would best serve national interests by returning to their medical studies with a view to early qualification, or service before qualification as surgeon-probationers in the Royal Navy.

The experience gained in this country confirms the views expressed at an early stage of the war by one at least of the large medical schools in London. War service for all medical students, *but service in a medical capacity*. All medical students should, for the sake of their friends, the public, and not least themselves, be enrolled in the medical section of an Officers' Training Corps; obtain certificates of satisfactory work and conduct from the authorities of their medical schools; and after passing the examination in anatomy and physiology, and holding some minor appointments in the wards of the hospital, they should serve, if required, for six months as surgeon-probationers in the Royal Navy, and then return to resume their work in the wards with a view to early qualification so as to re-join the Forces as medical officers.

The entry of medical students must proceed at a faster rate than that of a peace basis, for the loss of medical officers has been heavy. Women, however excellent in their work, will have always certain limitations of usefulness. The authorities of the medical schools can control the entry of male medical students, which can, if necessary, be checked by the returns for registration; the work of the students is shown by the results of the public examinations, and the early liability to service as a surgeon-probationer will deter any who might seek a "protected" calling. In any case it requires some courage to become a doctor, even in times of peace. If the war be a long one, the young medical students will do war service in a medical capacity; if the war be a short one, they will not be required as combatants at the early age at which boys now leave school to enter upon their medical studies.

The young boys are keen to serve, and sensitive to any charge of holding back. The authorities must, by their action, show that an industrious medical student is doing his country a greater service by training to serve her in a medical capacity.

